# Multiple System Atrophy-Cerebellar Variant With Absence of Parkinsonian Motor Features

**DOI:** 10.7759/cureus.110824

**Published:** 2026-06-14

**Authors:** Mohammed R Alasttal, Abdalrahman Alasmar, Wassem J Almadhoun, Nishat Ahmed

**Affiliations:** 1 Medicine and Surgery, Nasser Medical Complex, Gaza, PSE; 2 Medicine, Ungofaruu Hospital, Maldives, MDV; 3 Acute Medicine, Queen's Hospital Burton, Burton-on-Trent, GBR

**Keywords:** atypical parkinsonism, autonomic dysfunction, cerebellar ataxia, cerebellar degeneration, hot cross bun sign, msa-c, multiple system atrophy, neuroimaging, pontine atrophy, recurrent falls

## Abstract

Multiple system atrophy (MSA) is a rapidly progressive, multisystem, neurodegenerative disease characterized clinically by varying severity of Parkinsonian features, cerebellar, autonomic, and urogenital dysfunction, and corticospinal disorders. There are two subtypes of MSA - the Cerebellar and Parkinsonian types. The cerebellar subtype (MSA-C) commonly presents with gait ataxia, dysarthria, and coordination deficits; however, the absence of classical Parkinsonian features may delay diagnosis. We report the case of a 52-year-old man who presented with recurrent unexplained falls, progressive gait instability, dysarthria, and cerebellar signs without evidence of rigidity, resting tremor, or bradykinesia. The diagnosis of MSA-C was supported by neuroimaging, which showed significant pontocerebellar atrophy with the distinctive "hot cross bun" sign on MRI. Multiple conditions with similar progressive cerebellar symptoms, like spinocerebellar ataxias, Parkinson's disease, and progressive supranuclear palsy, may show overlapping manifestations. This shows the importance of thorough clinical assessment along with the distinctive neuroimaging in diagnosing MSA-C. In addition, better awareness of the clinical heterogeneity of MSA may facilitate earlier recognition and diagnosis.

## Introduction

Multiple system atrophy (MSA) is a rare, rapidly progressive neurodegenerative alpha-synucleinopathy characterized by autonomic dysfunction, Parkinsonism, cerebellar ataxia, and pyramidal signs [1,2]. It is characterized histopathologically by the presence of glial cytoplasmic inclusions containing aggregated α-synuclein, which cause neuronal degeneration involving the basal ganglia, cerebellum, brainstem, and autonomic nervous system [1,3]. MSA is categorically divided into two clinical phenotypes: the Parkinsonian subtype (MSA-P) and the cerebellar subtype (MSA-C) [2,4]. Although Parkinsonian features are frequently encountered in MSA, some patients initially present predominantly with cerebellar manifestations, making early diagnosis challenging [5,6].

The characteristic MRI finding, the "hot cross bun" sign, signifying pontocerebellar fiber degeneration, provides important radiological support for the diagnosis of MSA-C. However, it is not entirely specific and should be interpreted in conjunction with the overall clinical presentation [7,8]. Delayed recognition is common, especially in patients without classical extrapyramidal signs [5,9]. This report highlights the distinct presentation of MSA-C in a patient exhibiting progressive cerebellar impairment and repeated falls, occurring without evident Parkinsonian motor symptoms.

## Case presentation

A 52-year-old gentleman presented to the emergency department following an unwitnessed fall. The event was not observed, and the patient reported three similar falls within the preceding six months. He described these episodes as resulting from progressive unsteadiness while walking, without associated dizziness, lightheadedness, loss of consciousness, or identifiable precipitating factors.

Over the preceding year, he had also experienced a gradual slowing of mobility and progressive changes in speech, which had become increasingly slurred and heavy, according to both the patient and his relatives. He had attended the emergency department multiple times previously with similar falls, during which no major injuries or clear etiology had been identified, and the episodes were initially attributed to possible vasovagal attacks.

His past medical history included hypertension, asthma, type 2 diabetes mellitus, dyslipidemia, and autism spectrum disorder.

On examination, the patient was hemodynamically stable and generally well in appearance. Neurological examination revealed marked dysarthria with preserved cognition and orientation. There was no sensory deficit, and muscle tone, power, and deep tendon reflexes were all normal. There was no nystagmus. However, there were noticeable prominent cerebellar symptoms, including bilateral dysdiadochokinesia, dysmetria, intention tremor, diplopia, and an ataxic, unsteady gait. Notably, there was no evidence of resting tremor, rigidity, or bradykinesia. The remainder of the systemic examination was unremarkable.

Routine laboratory investigations and bedside assessments were within normal limits, and there was no evidence of orthostatic hypotension. However, during a subsequent admission, the patient reported urinary retention together with features of autonomic dysfunction, including impaired thermoregulation, emotional lability, and intermittent hyperhidrosis, raising further suspicion of underlying autonomic involvement. Computed tomography (CT) of the head demonstrated marked cerebellar atrophy with prominence of the cerebellar folia and fissures (Figure 1). 

**Figure 1:**
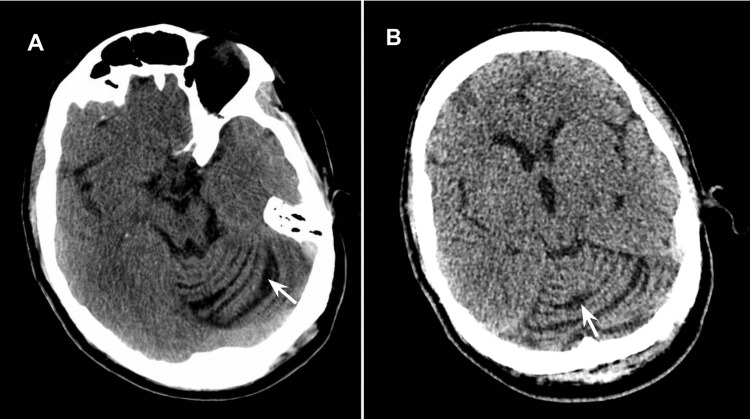
Non-contrast CT head demonstrating marked cerebellar atrophy A, B) axial CT images showing prominence of the cerebellar folia and widening of the cerebellar fissures (white arrows), consistent with significant cerebellar volume loss.

Further evaluation with magnetic resonance imaging (MRI) of the brain identified an incidental acute small-volume right cerebral convexity subdural hematoma. Neurosurgical assessment concluded that the lesion was not clinically significant and insufficient to explain the patient's symptoms. More importantly, MRI demonstrated pontine and cerebellar atrophy with the characteristic "hot cross bun" sign (Figure 2), highly suggestive of multiple system atrophy of the cerebellar subtype (MSA-C). Despite MSA being classified among atypical Parkinsonian syndromes, the patient did not exhibit classical Parkinsonian motor manifestations. 

**Figure 2:**
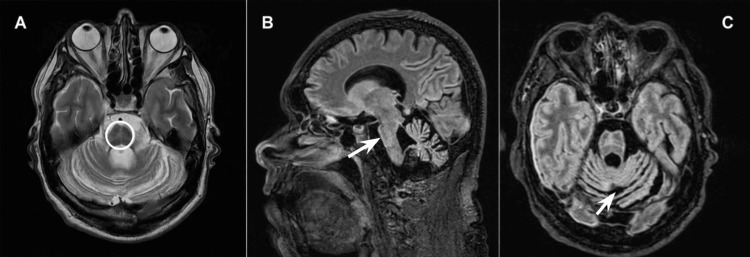
MRI brain demonstrating characteristic radiological features of MSA-C A) Axial T2-weighted image showing the characteristic "hot cross bun" sign within the pons (white circle); B) Sagittal image demonstrating pontine atrophy (white arrow); C) Axial image showing cerebellar atrophy with prominence of the cerebellar folia and widening of the cerebellar fissures (white arrow). MSA-C - multiple system atrophy cerebellar subtype

## Discussion

Multiple system atrophy is a progressive adult-onset neurodegenerative disorder characterized by varying degrees of autonomic dysfunction, cerebellar ataxia, Parkinsonism, and pyramidal involvement [1,2]. MSA is clinically divided into two major phenotypes: MSA-P, in which Parkinsonian features predominate, and MSA-C, which is characterized mainly by cerebellar dysfunction [2,4]. Geographic variation in phenotype distribution has been well described. European studies reported a predominance of MSA-P, accounting for approximately 68% of cases [10], whereas Japanese cohorts demonstrated a higher frequency of MSA-C, representing nearly 84% of patients [11].

This case is notable because the patient presented with progressive cerebellar dysfunction without clear Parkinsonian signs such as rigidity, resting tremor, or bradykinesia. Such presentations may delay recognition and can initially mimic other causes of cerebellar degeneration, including hereditary spinocerebellar ataxias, alcohol-related cerebellar disease, autoimmune cerebellar syndromes, vascular pathology, paraneoplastic disorders, and sporadic adult-onset ataxia [5,6,12]. In this patient, the absence of a relevant family history, sensory deficits, or radiological evidence of vascular pathology, together with the characteristic MRI findings and subsequent development of urinary retention suggestive of autonomic involvement, ultimately favored a diagnosis of MSA-C.

Neuroimaging played a key role in narrowing the diagnosis. MRI demonstrated pontine and cerebellar atrophy together with the characteristic "hot cross bun" sign, reflecting degeneration of pontocerebellar fibers [7,8,13]. Although not specific to MSA-C, this finding strongly supports the diagnosis when interpreted alongside compatible clinical features.

Autonomic dysfunction is considered a core feature of MSA, although symptoms may be minimal or absent early in the disease course [1,14]. Therefore, the absence of orthostatic hypotension in this patient does not exclude the diagnosis. However, the patient subsequently presented to the emergency department (ED) with urinary retention, suggesting evolving autonomic involvement as the disease progressed.

Management remains largely supportive, as no disease-modifying therapy is currently available [1,15]. Treatment focuses on symptom control and multidisciplinary rehabilitation involving physiotherapy, occupational therapy, speech and language therapy, nutritional support, and management of autonomic complications [15,16]. In this case, following the diagnosis of MSA-C, the patient was referred to a specialist movement disorders clinic and received physiotherapy and occupational therapy input as part of his ongoing management. Physiotherapy and gait rehabilitation are especially important in reducing fall risk and maintaining mobility.

Pharmacological therapy may help selected symptoms, particularly autonomic dysfunction. Dopaminergic treatment can provide limited benefit in some patients, although the response in MSA-C is generally less pronounced than in idiopathic Parkinson's disease [1,17]. Experimental therapies targeting α-synuclein accumulation and neuroinflammation remain under investigation [15,17].

This case highlights the diagnostic challenge of cerebellar-predominant MSA, especially in patients without obvious Parkinsonian or autonomic manifestations at presentation. Early recognition is important to facilitate supportive management, rehabilitation, counseling, and long-term care planning.

## Conclusions

This case illustrates the diagnostic complexity of cerebellar-predominant multiple system atrophy, especially when the first manifestation lacks significant Parkinsonian characteristics. This patient exhibited progressive gait instability, frequent falls, and cerebellar dysfunction without conventional extrapyramidal symptoms, resulting in significant diagnostic ambiguity in the early disease stages. This example underscores the significance of identifying distinctive radiological abnormalities, particularly the "hot cross bun" sign, when assessed in conjunction with relevant clinical symptoms. When evaluating patients with progressive cerebellar syndromes, physicians should consider a broad spectrum of potential aetiologies, as autonomic and Parkinsonian manifestations may initially be ambiguous or incomplete. Prompt recognition of unusual MSA-C manifestations may enhance timely supportive management, multimodal rehabilitation, suitable counseling, and improved long-term care planning for impacted individuals.
